# Enzymatically synthesized glycogen inhibited degranulation and inflammatory responses through stimulation of intestine

**DOI:** 10.3164/jcbn.20-33

**Published:** 2020-06-05

**Authors:** Yasukiyo Yoshioka, Masako Inoue, Hiroko Yoshioka, Tomoya Kitakaze, Takashi Furuyashiki, Naoki Abe, Hitoshi Ashida

**Affiliations:** 1 Department of Clinical Nutrition and Dietetics, Faculty of Clinical Nutrition and Dietetics, Konan Women’s University, 6-2-23 Morikita-machi, Higashinada-ku, Kobe 658-0001, Japan; 2 Graduate School of Science, Technology and Innovation, Kobe University, 1-1 Rokkodai-cho, Nada-ku, Kobe 657-8501, Japan; 3 Department of Agrobioscience, Graduate School of Agricultural Science, Kobe University, 1-1 Rokkodai-cho, Nada-ku, Kobe 657-8501, Japan; 4 Department of Food Science and Nutrition, School of Human Environmental Sciences, Mukogawa Women’s University, 6-46 Ikebiraki-cho, Nishinomiya, Hyogo 663-8558, Japan; 5 Institute of Health Sciences, Ezaki Glico Co., Ltd., 4-6-5 Utajima, Nishiyodogawa-ku, Osaka 555-8502, Japan; 6 Department of Nutritional Science and Food Safety, Faculty of Applied Science, Tokyo University of Agriculture, 1-1-1 Sakuragaoka, Setagaya-ku, Tokyo 156-8502, Japan

**Keywords:** enzymatically synthesized glycogen, anti-allergy, anti-inflammation, Caco-2 cells, RBL-2H3 cells

## Abstract

The patients of type I allergic diseases were increased in the developed countries. Recently, many studies have focused on food factors with anti-allergic activities. Enzymatically synthesized glycogen, a polysaccharide with a multi-branched α-1,4 and α-1,6 linkages, is a commercially available product from natural plant starch, and has immunostimulation activity. However, effect of enzymatically synthesized glycogen on the anti-allergic activity was unclear yet. In this study, we investigated that enzymatically synthesized glycogen inhibited allergic and inflammatory responses using a co-culture system consisting of Caco-2 and RBL-2H3 cells. Enzymatically synthesized glycogen inhibited antigen-induced β-hexosaminidase release and production of TNF-α and IL-6 in RBL-2H3 cells in the co-culture system. Furthermore, enzymatically synthesized glycogen inhibited antigen-induced phosphorylation of tyrosine kinases, phospholipase C γ1/2, mitogen-activated protein kinases and Akt. Anti-allergic and anti-inflammatory activities of enzymatically synthesized glycogen were indirect action through stimulating Caco-2 cells, but not by the direct interaction with RBL-2H3 cells, because enzymatically synthesized glycogen did not permeate Caco-2 cells. These findings suggest that enzymatically synthesized glycogen is an effective food ingredient for prevention of type I allergy through stimulating the intestinal cells.

## Introduction

Allergic diseases are divided into four types. Of these, type I allergic diseases are a major public health problem all over the world. Type I allergic diseases are induced by certain allergens such as certain food ingredients, dust, and pollen.^(1)^ The fundamental approaches of therapy for type I allergic diseases have not been clarified. Activation of mast cells trigger allergic and inflammatory responses through the release of mediators such as histamine, inflammatory cytokines and arachidonic acid metabolites and the produced cytokines and chemokines cause immune responses in turn.^(2,3)^ A remarkable mechanism for the activation of mast cells is crosslinking of FcɛRI, the high-affinity receptor for immunoglobulin E (IgE), by a multivalent antigen. Antigen binds to the cell-associated IgE and triggers degranulation of the mast cells or basophils, causing the release of their allergy-producing mediators and also the sustained synthesis and release of certain cytokines, chemokines and growth factors.^(4)^ Tumor necrosis factor (TNF)-α is one of the multifunctional cytokines that mediates various inflammation-promoting biologic activities.^(5)^ Thus, the inhibitors of degranulation or TNF-α production in activated mast cells and basophils are possible to be a candidate for anti-allergic food ingredient.

Glycogen, a multi-branched α-1,4 and α-1,6 linkages, is the major form of energy storage in animals, plants, and fungi.^(6)^ Enzymatically synthesized glycogen (ESG) is produced from plant starch.^(7)^ The molecular structure of ESG is different from that of natural glycogen. α-1,6 Linkages in ESG molecule are buried in the center of molecule and its linkages form a huge cluster, whereas those in the natural glycogen are distributed near the surface of molecule.^(8)^ Thus, ESG molecules are partially digested with α-amylase and the undigested part of ESG is named resistant glycogen (RG).^(9)^ After the oral administration of ESG in rodents, it has been reported that the glycemic index of ESG is about 80%, indicating that 20% of the ESG is remained as indigestible RG.^(10)^ Previously, ESG has been reported to possess certain biological effects such as immunostimulation activity, promotion of osteogenesis, inhibition of colitis, and prevention of metabolic disorders.^(10–14)^ In addition, ESG-induced immunomodulatory activity was observed without permeation of intestine.^(15)^ Therefore, to estimation the function of EGS *in vitro*, a co-culture system using intestinal cells and target tissue ones is an useful tool.

In the present study, we evaluated anti-allergic effects of ESG using the co-culture system, in which Caco-2 and basophils or mast cells were plated on the apical and the basolateral sides, respectively, and measured an activity of β-hexosaminidase released from antigen-induced basophils or mast cells as an index of allergy activity. Furthermore, production of TNF-α and IL-6 as the inflammatory cytokines and its upstream events were investigated to understand the inhibitory mechanism of ESG against allergy and inflammation responses.

## Materials and Methods

### Reagents

ESG was prepared from plant starch as a substrate using three enzymes as previously described.^(14)^ The molecular weight of ESG is approximately 8,700 kDa.^(15)^ As a hapten-conjugated protein and its anti-body, dinitrophenylated bovine serum albumin (DNP-BSA) and mouse anti-dinitrophenyl (DNP) IgE were purchased from Calbiochem (San Diego, CA) and Sigma Chemical (St. Louis, MO), respectively. Antibodies against Akt, phosphorylated Akt, Lyn, phosphorylated Lyn, Syk, phosphorylated Syk, phospholipase C (PLC) γ1/2, phosphorylated PLCγ1/2, p38, phosphorylated p38, JNK, phosphorylated JNK and β-actin were purchased from Cell Signaling Technology Co. (Denvers, MA). Antibodies against TNF-α and IL-6 were from Abcam (Cambridge, UK). Horseradish peroxidase (HRP)-conjugated anti-rabbit IgG was from Bio-Rad Laboratories Inc. (Hercules, CA). Blocking One, and Blocking One-P solutions were from Nacalai Tesque, Inc. (Kyoto, Japan). Polyvinylidene difluoride membrane was from GE Healthcare (Fairfield, WA). Immunostar^®^ LD chemiluminescence detection kit was a product of FUJIFILM Wako Pure Chemical Corporation (Osaka, Japan). All other reagents were of the highest grade commercially available.

### Cell culture

Rat basophilic leukemia cell linage, RBL-2H3,^(16)^ was maintained and cultures in Dulbecco’s modified Eagle’s medium (DMEM) supplemented with 10% (v/v) fetal bovine serum, 2 mM l-glutamine, 100 µg/ml streptomycin, 100 U/ml penicillin in an incubator with 5% CO_2_ at 37°C, while human intestinal epithelial cell linage, Caco-2, was in DMEM supplemented with 10% (v/v) fetal bovine serum, 4.5 g/L glucose, 1% non-essential amino acids, 2 mM l-glutamine, 100 µg/ml streptomycin, 100 U/ml penicillin. For differentiation, Caco-2 cells were incubated for 2 weeks. The formation of the Caco-2 monolayer was assessed by measuring transepithelial electrical resistance (TEER) value. Tight junctions act as a barrier to paracellular diffusion and TEER reflects the tightness of junctions between epithelial cells.^(17,18)^ Monolayer cells were gently rinsed with Hank’s balanced salt solution (HBSS) containing 137 mM NaCl, 5.36 mM KCl, 1.26 mM CaCl_2_, 0.55 mM MgSO_4_, 0.44 mM KH_2_PO_4_, 0.34 mM Na_2_HPO_4_, 2.92 mM NaH_2_PO_4_ and incubated for 30 min in an incubator. The formation of the cell monolayer was evaluated by measuring TEER values using Millicell-ERS (Millipore, Eschborn, Germany). Bone marrow-derived mast cells (BMMC) were prepared from BALB/c male mice (8-weeks old) and maintained in RPMI1640 medium (Gibco, Grand Island, NY) supplemented with 10% fetal bovine serum, 2 mM l-glutamine, 100 µg/ml streptomycin, 100 U/ml penicillin, 5 ng/ml recombinant murine IL-3 (Peprotech, Rocky Hill, NJ) and 10 ng/ml recombinant murine stem cell factor (Peprotech) as described previously.^(19)^

### Degranulation assay

RBL-2H3 cells or BMMC were harvested in a 24-well culture plate and sensitized with 1 µg/ml anti-DNP IgE for 16 h at 37°C. After washing with PIPES buffer, pH 7.2 containing 119 mM NaCl, 5 mM KCl, 0.4 mM MgCl_2_, 1 mM CaCl_2_, 40 mM NaOH, 25 mM PIPES, 5.6 mM glucose, and 0.1% BSA, the cells were incubated with several concentrations of ESG for 24 h at 37°C. After the incubation, the cells were challenged with 10 ng/ml DNP-BSA for 30 min at 37°C. The plate was cooled on the ice to stop degranulation response. The supernatant was transferred to a new tube and incubated with a substrate solution consisting of 0.2 M citrate, pH 4.5 containing 5 mM *p*-nitrophenyl-*N*-acetyl-β-d-glucosaminide for another 1 h at 37°C. The reaction was stopped by adding 0.2 M glycine, pH 10.0, and the absorbance at 405 nm was measured using a spectrophotometer U-3900 (HITACHI, Japan). Percent of β-hexosaminidase released into the supernatant was calculated.

### Caco-2/RBL-2H3 cells and Caco-2/BMMC co-culture systems

Caco-2 cells were seeded on a 24-well Transwell insert plate (0.33 cm^2^, 0.4 µm pore size, Corning Costar Corp., Cambridge, MA). The formation of the Caco-2 monolayer was assessed by measuring TEER values using Millicell-ERS. The formation of monolayer was confirmed that TEER value was over 300 Ω·cm^2^. RBL-2H3 cells or BMMC were seeded on the 24-well tissue culture plate and incubated overnight. The Transwell insert, on which Caco-2 cells had been cultured, was set to the well on the plate and pre-cultured RBL-2H3 cells or BMMC. ESG was added to the apical side and incubated for 24 h. After incubation, RBL-2H3 cells or BMMC in the basolateral side were sensitized with 1 µg/ml anti-DNP IgE for 3 h. RBL-2H3 cells or BMMC were washed with PIPES buffer and challenged with 10 ng/ml DNP-BSA for another 30 min. The plate was cooled on the ice to stop degranulation response. Measurement of degranulation, β-hexosaminidase activity, performed as described above.

### Western blotting

RBL-2H3 cells from the Caco-2/RBL-2H3 co-culture system were lysed with RIPA buffer, pH 8.0 containing 50 mM Tris, 150 mM sodium chloride, 1% (v/v) NP-40, 0.5% (w/v) deoxycholic acid, 0.1% (w/v) sodium dodecyl sulfate, 0.5 mM dithiothreitol, protease inhibitors (1 mM phenylmethylsulfonyl fluoride, 5 µg/ml leupeptin and 5 µg/ml aprotinin), and phosphatase inhibitors [10 mM sodium fluoride and 1 mM sodium orthovanadate (V)]. The lysate was centrifuged at 20,000 × *g* for 20 min and the supernatant was used as the cell lysate. The cell lysate was mixed with sodium dodecyl sulfate sample buffer consisting of 62.5 mM Tris, pH 6.8, 2% (w/v) sodium dodecyl sulfate, 10% (v/v) glycerol, 5% (v/v) 2-mercaptoethanol, and 0.02% (w/v) bromophenol blue. The mixture was incubated at 100°C for 5 min and subjected to sodium dodecyl sulfate-polyacrylamide gel electrophoresis. The separated proteins in the gels were transferred onto a polyvinylidene fluoride membrane. The membrane was incubated with a blocking solution consisting of Blocking One (for detection of unphosphorylated proteins) or Blocking One-P (for detection of phosphoproteins) for 1 h at room temperature and treatment with primary antibodies overnight at 4°C, followed by the corresponding horseradish peroxidase-conjugated secondary antibody for another 1 h at room temperature. Protein bands were visualized using Immuno Star^®^ LD Western Blotting Substrate and detected with Light-Capture II (ATTO, Tokyo, Japan). The density of the specific band was determined using ImageJ image analysis software (National Institutes of Health, Bethesda, MD).

### Enzyme-linked immunosorbent assay (ELISA)

To determine the concentration of ESG in medium from the both apical and basolateral sides of the Caco-2/RBL-2H3 cells co-culture system, ELISA was performed as follows: ESG at 1,000 µg/ml was applied to Caco-2 monolayers on the Transwell inserts in the 24-well plate for 24 h. Medium of the apical and basolateral sides was separately collected, diluted 1,000 times, and subjected to ELISA. Diluted medium and several concentrations of ESG were coated onto the 96-well micro titer plate for 24 h at 4°C and blocking non-specific binding using Blocking One for 1 h at 37°C. As a prime antibody, conditioned medium of hybridoma producing ESG1A9mAb^(9)^ was added to the well and incubated for 2 h at 37°C. As a secondary antibody, horse radish peroxidase-labeled mouse anti-IgM antibody was used. The bound antibodies were visualized using TMB reagent (DAKO, Glostrup, Denmark) and the reaction was terminated by addition of 1 M H_2_SO_4_. Between each step, the well was washed three times with PBS containing 0.05% Tween 20. The absorbance at 450 nm was measured using a multi-labeled palate reader Wallac 1420 ARVOSX (Perkin Elmer. Co., Waltham, MA).

### Statistical analysis

Statistical analysis was performed with JMP statistical software ver. 11.2.0 (SAS Institute. Cary, NC). Data are represented as the mean ± SE. The statistical significance of experimental observations was determined using Dunnett’s test and Tukey-Kramer test. The level of significance was set as *p*<0.05.

## Results

### ESG inhibited degranulation from RBL-2H3 cells and BMMC in the Caco-2/RBL-2H3 cells and Caco-2/BMMC co-culture systems

**Fig. 1:**
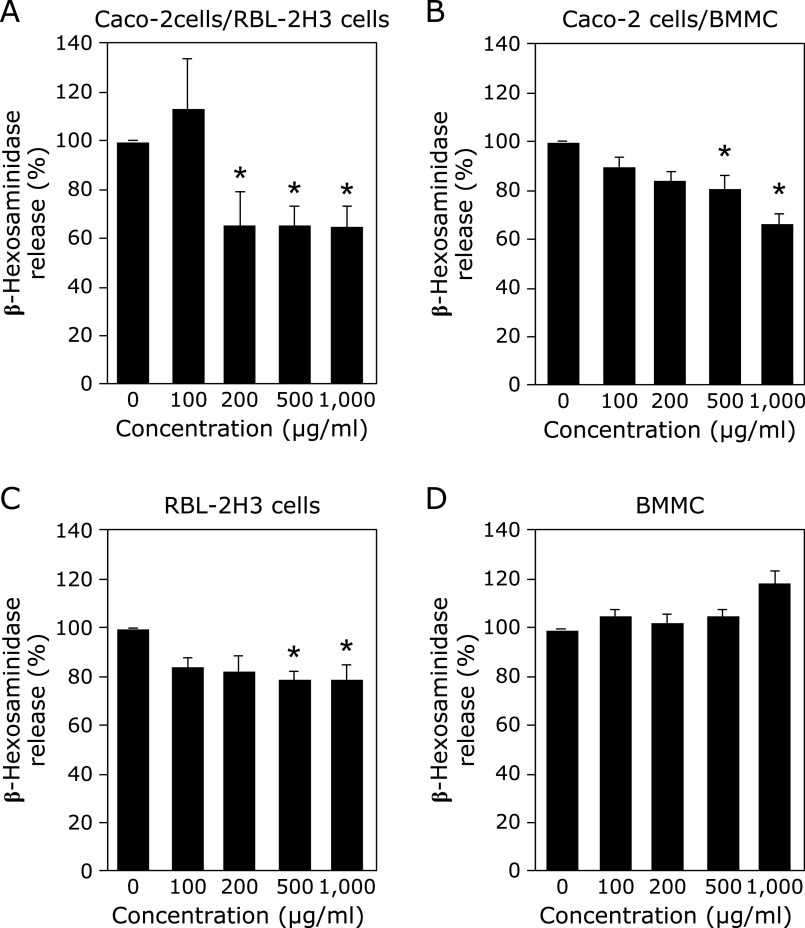
Inhibitory effects of ESG on β-hexosaminidase release. Caco-2 cells were seeded to a 24-well Transwell insert plate and fully differentiated. RBL-2H3 cells (A) or BMMC (B) were seeded to the 24-well tissue culture plate and incubated overnight. ESG was added to Caco-2 cells in the apical side and incubated for 24 h. After incubation, RBL-2H3 cells or BMMC in the basolateral side were stimulated with anti-DNP-IgE/DNP-BSA. RBL-2H3 cells (C) or BMMC (D) were seeded in the 24-well tissue plate and incubated overnight. ESG was directly added to RBL-2H3 cells or BMMC and incubated for 24 h. After incubation, RBL-2H3 cells or BMMC were stimulated with anti-DNP-IgE/DNP-BSA. The medium was incubated with a *p*-nitrophenyl-*N*-acetyl-β-d-glucosaminide for 1 h at 37°C. The absorbance at 405 nm was measured. Data are presented as the mean ± SE (*n* = 3). *Significant difference from the β-hexosaminidase activity in the supernatant from the corresponding control cells by Dunnett’s test (*p*<0.05).

First, it was examined whether ESG inhibited degranulation from RBL-2H3 cells in the co-culture system. Stimulation of DNP-BSA to DNP-specific IgE-primed RBL-2H3 cells resulted in β-hexosaminidase release from the cells. Treatment with ESG to Caco-2 cells inhibited the β-hexosaminidase release from RBL-2H3 cells in dose-dependent manner (Fig. 1A). Significant inhibition was observed ESG at 200 µg/ml and higher concentration.

It was also examined inhibitory effect of ESG against degranulation from BMMC in the co-culture system. BMMC is mast cells differentiated from mouse bone marrow stem cells and is non-cancerous cells. Stimulation of DNP-BSA to DNP-specific IgE-primed BMMC resulted in β-hexosaminidase release from the cells. Treatment with ESG at 500 and 1,000 µg/ml to Caco-2 cells inhibited the β-hexosaminidase release from BMMC (Fig. 1B). These results indicated that ESG inhibited degranulation in both basophiles and mast cells after co-cultured with Caco-2 cells.

### Direct inhibitory effect of ESG on degranulation from RBL-2H3 cells and BMMC

Direct inhibitory effect of ESG on degranulation from basophils and mast cells was further investigated. When ESG was treated with RBL-2H3 cells and BMMC under the same concentration range as Fig. 1, treatment with ESG at 500 and 1,000 µg/ml slightly inhibited antigen-stimulated degranulation in RBL-2H3 cells (Fig. 1C). However, the inhibition extent was lower than the co-culture with Caco-2 cells. In addition, ESG did not affect degranulation from BMMC after the direct treatment with ESG (Fig. 1D).

### The inhibitory mechanism of ESG against antigen-stimulated degranulation in the Caco-2/RBL-2H3 cells co-culture system

**Fig. 2:**
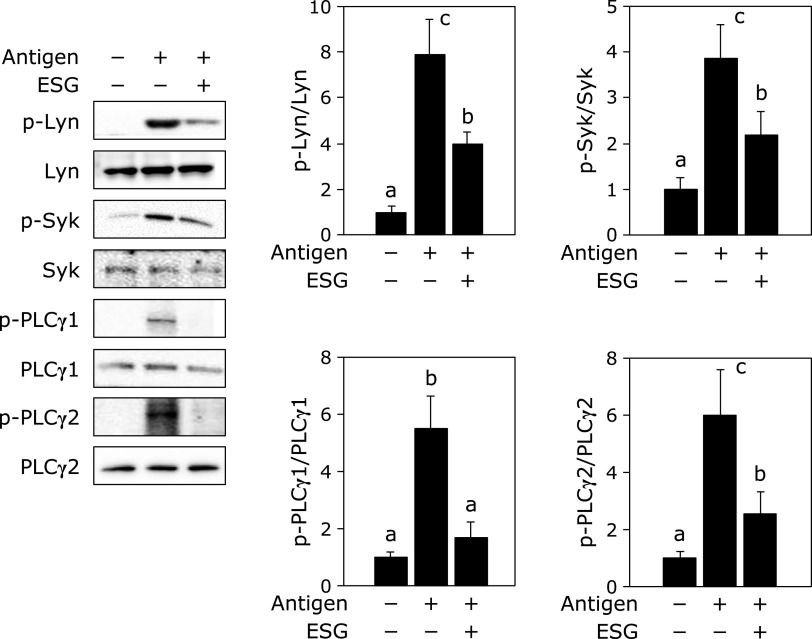
ESG inhibited phosphorylation of the upstream mediators of degranulation in RBL-2H3 cells co-cultured with Caco-2 cells. Co-culture of RBL-2H3 cells with Caco-2 cells was performed as the same procedure as described in Fig. 1. After ESG was treated to the Caco-2 cells in the apical side for 24 h, RBL-2H3 cells in the basolateral side were stimulated with anti-DNP-IgE/DNP-BSA. The cell lysate from RBL-2H3 cells was subjected to western blotting. Data are presented as the mean ± SE (*n* = 3). Different letters indicate significant differences by Tukey-Kramer test (*p*<0.05).

To elucidate the inhibitory mechanism of ESG against antigen-stimulated degranulation in the Caco-2/RBL-2H3 cells co-culture system, the expression and phosphorylation levels of intracellular mediators related to degranulation were investigated. As expected, stimulation of antigen increased in the phosphorylation level of degranulation mediators, Lyn, Syk and PLCγ1/2 in RBL-2H3 cells. Treatment with ESG at 1,000 µg/ml to Caco-2 cells significantly inhibited antigen stimulation-increased phosphorylation level of these mediators without affecting their expression levels in RBL-2H3 cells (Fig. 2).

### Treatment with ESG to Caco-2 cells inhibited inflammatory cytokine production in the Caco-2/RBL-2H3 cells co-culture system

**Fig. 3:**
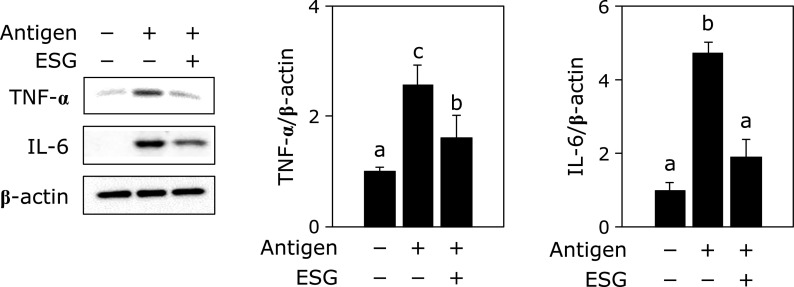
ESG inhibited the production of TNF-α and IL-6 in RBL-2H3 cells co-cultured with Caco-2 cells. Co-culture of RBL-2H3 cells with Caco-2 cells was performed as the same procedure as described in Fig. 1. After ESG was treated to the Caco-2 cells in the apical side for 24 h, RBL-2H3 cells in the basolateral side were stimulated with anti-DNP-IgE/DNP-BSA. The cell lysate from RBL-2H3 cells was subjected to western blotting. Data are presented as the mean ± SE (*n* = 3). Different letters indicate significant differences by Tukey-Kramer test (*p*<0.05).

**Fig. 4:**
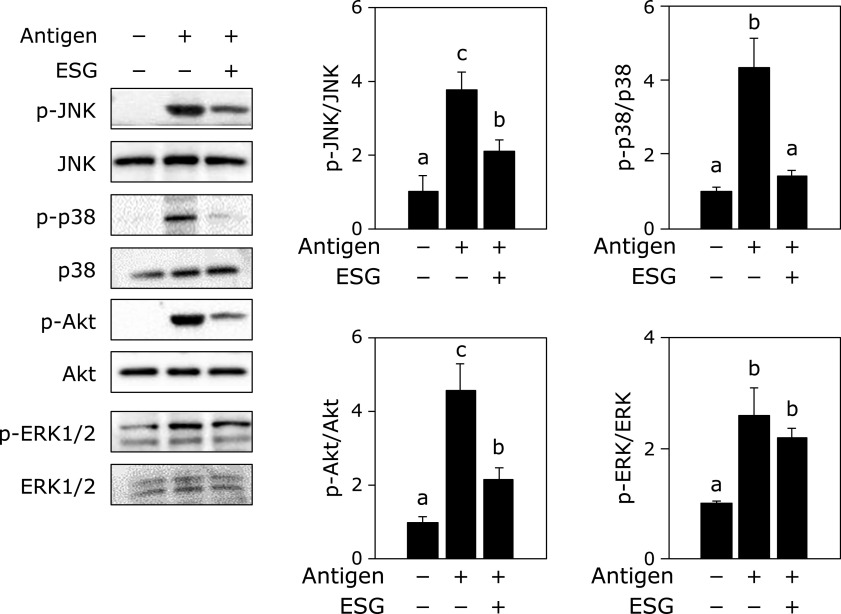
ESG inhibited phosphorylation of the upstream mediators of inflammatory cytokine productions in RBL-2H3 cells co-cultured with Caco-2 cells. Co-culture of RBL-2H3 cells with Caco-2 cells was performed as the same procedure as described in Fig. 1. After ESG was treated to the Caco-2 cells in the apical side for 24 h, RBL-2H3 cells in the basolateral side were stimulated with anti-DNP-IgE/DNP-BSA. The cell lysate from RBL-2H3 cells was subjected to western blotting. Data are presented as the mean ± SE (*n* = 3). Different letters indicate significant differences by Tukey-Kramer test (*p*<0.05).

Since ESG inhibited antigen-stimulated degranulation from basophils and mast cells in the co-culture with Caco-2 cells, further experiment was performed on the protein expression levels of inflammatory cytokines in RBL-2H3 cells. Treatment with ESG at 1,000 µg/ml to Caco-2 cells significantly inhibited antigen stimulation-increased TNF-α and IL-6 production in RBL-2H3 cells (Fig. 3). It was reported that JNK, p38 and Akt are involved in the production of TNF-α and IL-6 as the downstream factors for Syk.^(20)^ Their phosphorylation levels were also investigated. Treatment with ESG at 1,000 µg/ml to Caco-2 cells inhibited antigen stimulation-increased in the phosphorylation of JNK, p38 and Akt, but not ERK1/2, in RBL-2H3 cells (Fig. 4).

### ESG did not permeate through Caco-2 cells

**Fig. 5:**
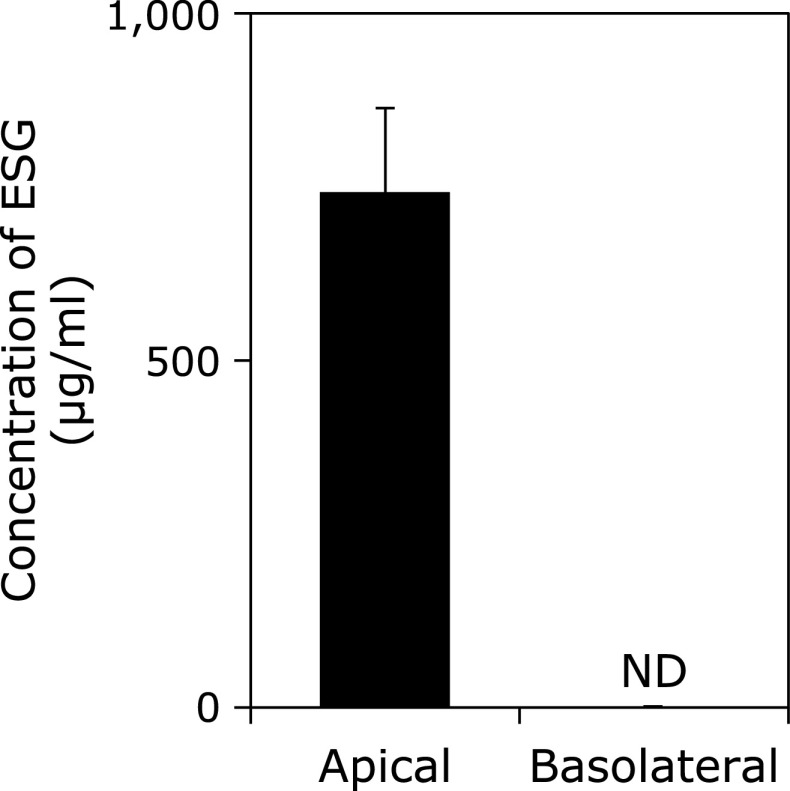
ESG did not penetrate Caco-2 cells. Co-culture of RBL-2H3 cells with Caco-2 cells was performed as the same procedure as described in Fig. 1. ESG at 1,000 µg/ml was applied to Caco-2 monolayers in the apical side for 24 h. The medium in the apical and basolateral sides were collected. The concentration of ESG in both supernatants was measured by ELISA as described in the Materials and Methods section.

Finally, it was investigated whether ESG can permeate Caco-2 cells in the co-culture system. The concentration of ESG in both apical and basolateral sides in Caco-2/RBL-2H3 cells co-culture system was measured using ELISA system. After treatment with ESG at 1,000 µg/ml to the apical side, ESG could not detect in the basolateral side, though about 700 µg/ml of ESG was remained in the apical side (Fig. 5). These results strongly suggest that ESG cannot permeate Caco-2 cells.

## Discussion

Patients affecting with type I allergy such as food allergy, allergic rhinitis, asthma and atopic dermatitis has markedly increased in developed countries. Certain factors in food, environment and genetics are known to impact on development of type I allergy.^(1)^ It is an important concern that the factors increase in prevalence of type I allergy.^(21)^ Various food factors are reported possess anti-allergic activities and examined as potential resources for their therapeutics.^(22)^ For examples, Epigallocatechin gallate inhibits histamine release through suppression of FcɛRI expression and Ca^2+^ influx in RBL-2H3 cells.^(23)^ Resveratrol inhibits IgE-mediated basophilic mast cell degranulation through suppression of protein kinase C activation.^(24)^ To investigate the inhibitory effect of food factors against the activation of mast cells such as degranulation and inflammation response, most studies have been used RBL-2H3 cells.^(25,26)^ Many studies has been demonstrated the results of degranulation and inflammatory cytokine production after direct treatment with food factors to RBL-2H3 cells.^(27–30)^ Orally administered food factors can not directly interact with immune cells and requires permeation to the intestinal tract. The intestinal epithelium is the first barrier against antigens and intermittently exposed to antigens in the form of food, dust, and pollen.^(31)^ The co-culture system consisting of the intestinal cells and basophils or mast cells is suitable method for evaluation of anti-allergy activity. Indeed this system has been used for estimation of the anti-allergy activity of certain food factors.^(32,33)^

**Fig. 6:**
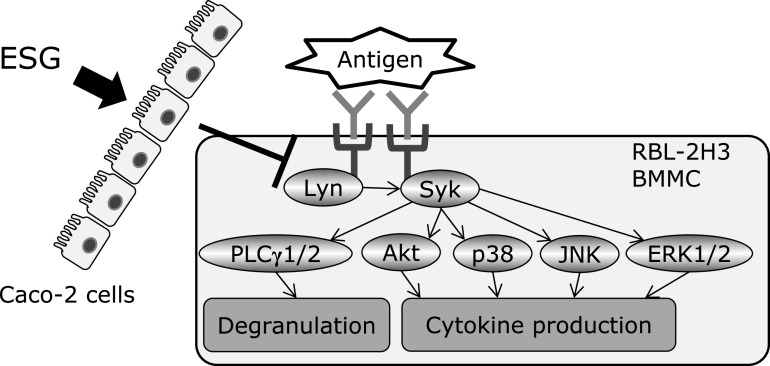
Putative molecular mechanism of ESG for anti-allergy and anti-inflammatory response.

In this study, we demonstrated that ESG inhibited allergic and inflammatory responses using co-culture system with Caco-2 and RBL-2H3 or BMMC. Treatment with ESG inhibited antigen-induced β-hexosaminidase release, a marker of histamine release, from RBL-2H3 or BMMC co-cultured with Caco-2 cells (Fig. 1). ESG also inhibited antigen-induced inflammatory cytokines, TNF-α and IL-6 (Fig. 3). As underlying molecular mechanism in co-cultured RBL-2H3 cells with Caco-2 cells, ESG inhibited antigen-induced phosphorylation of Lyn and Syk, and PLCγ1/2 that are upstream regulators for histamine release (Fig. 2). Furthermore, ESG inhibited JNK, p38, and Akt (Fig. 4). It has been reported that PLCγ1/2 is regulated by phosphoinositide 3-kinase.^(34,35)^ It has been also reported that LY294002, a specific inhibitor of phosphoinsitide 3-kinase, blocked the degranulation, but mitogen-activated protein kinase activation is no effect.^(36)^ Activation of ERK and JNK mitogen-activated protein kinases has been reported as Syk activation-dependent in FcɛRI-mediated pathway.^(20)^ Akt is regulated by Lyn/Syk signaling.^(37)^ These observations indicated that phosphorylation of Syk is essential for the degranulation and inflammatory cytokine production signal transduction. Thus, ESG inhibited degranulation and cytokine production through suppression of Syk activation in RBL-2H3 cells co-cultured with Caco-2 cells. Putative molecular mechanism of ESG for anti-allergy and anti-inflammatory response is illustrated in Fig. 6.

Since ESG is a macromolecule, it difficult permeates intestinal cells. Indeed, we confirmed ESG did not permeate Caco-2 cells (Fig. 5). It was noteworthy that, inhibition of antigen-induced β-hexosaminidase release was attenuated by direct treatment with ESG to RBL-2H3 and was not observed in BMMC (Fig. 1C and D). Previously, ESG has reported to partially degrade to RG, which activated immune cells by stimulating the intestinal cells without permeation of the intestinal barrier.^(15)^ ESG has reported to be bound to Toll-like receptor 2 (TLR2) and activates signaling molecules via TLR2, resulting in the stimulation of immune cells.^(38)^ TLR2 is expressing in various human intestinal cells including Caco-2 cells. However, ESG inhibited β-hexosaminidase release from RBL-2H3 cells in the co-culture with TLR2-knock downed Caco-2 cells (data not shown). Thus, TLR2 is not involved in the anti-allergic activity of ESG. Therefore, it is suggested that certain unknown factor secreted from Caco-2 cells is involved in the ESG-caused inhibition of antigen-induced β-hexosaminidase. Further study is needed to identify the secreted factor from the intestinal cells for the anti-allergy and anti-inflammatory activities.

In conclusion, ESG inhibited antigen-induced β-hexosaminidase release and production of TNF-α and IL-6 from RBL-2H3 cells in the co-culture system with Caco-2 cells. As the mechanism, ESG attenuated antigen-induced phosphorylation of Lyn and Syk, PLCγ1/2, JNK, p38, and Akt in RBL-2H3 cells. These findings suggested that ESG is an effective food ingredient for prevention of type I allergy.

## Abbreviations

BMMC: bone marrow-derived mast cells
DMEM: Dulbecco’s modified Eagle’s medium
DNP: dinitrophenyl
DNP-BSA: dinitrophenylated bovine serum albumin
ELISA: enzyme-linked immunosorbent assay
ESG: enzymatically synthesized glycogen
HBSS: Hank’s balanced salt solution
IgE: immunoglobulin E
PLC: phospholipase C
RG: resistant glycogen
TEER: transepithelial electrical resistance
TLR: Toll-like receptor
TNF: tumor necrosis factor
